# Fluorescent Paper Based on CQDs/Rhodamine B: A Ratio and Sensitive Detection Platform for On-Site Fe^3+^ Sensing

**DOI:** 10.3390/molecules29071658

**Published:** 2024-04-07

**Authors:** Guangda Han, Jihai Cai, Lu Yang, Xiaoyun Li, Xiaoying Wang

**Affiliations:** 1 State Key Laboratory of Pulp & Paper Engineering, South China University of Technology, Wushan Road, Tianhe District, Guangzhou 510640, China; 18309292176@163.com (G.H.); jihaicai@scut.edu.cn (J.C.); lxy26@scut.edu.cn (X.L.); 2 Xi’an Rare Metal Materials Institute Co., Ltd., Xi’an 710016, China; 3 School of Civil Aviation, Northwestern Polytechnical University, Xi’an 710072, China; 18700835199@163.com

**Keywords:** ratio fluorescence paper, smartphone, carbon quantum dots, Fe^3+^ sensing, xylan

## Abstract

Fluorescent sensors with single reading are generally subject to unpredictable disturbs from environmental and artificial factors. In order to overcome this barrier of detection reliability, a paper-based optical sensor with proportional fluorescence was established and further combined with a smartphone for visual, on-site and quantitative detection of Fe^3+^, which affects the color, smell and taste of water, and endangers the health of plants and animals. The ratio fluorescent probe was fabricated by rhodamine B and carbon quantum dots derived from xylan. The red fluorescence of rhodamine B was inert to Fe^3+^, which was referred to as background. And blue emitting carbon quantum dots functioned as signal report units, which would be quenched by Fe^3+^ and make the fluorescence of the ratio probe change from purple to red. The quantitative detection of Fe^3+^ was conducted by investigating the RGB value of fluorescent images with a smartphone. With the increase of Fe^3+^ concentration, the R/B (red/blue) value of the fluorescent paper gradually increased. The linear detection range was 10–180 μM, and the limit of detection was 198.2 nM. The application of ratio fluorescent paper with a smartphone provides a facile method for the rapid detection of ions.

## 1. Introduction

Iron is an important metal element in life, smelting and industry, and plays a very important role in the progress of material civilization. However, when the concentration of Fe^3+^ in water is 0.1 to 0.3 mg/L, it will affect the color, smell and taste of water. Usually, water has the function of self-purification, but when the amount of Fe^3+^ reaches a certain level, the water body will not be able to self-purify, which will destroy the ecological environment [1,2]. Therefore, the monitoring of Fe^3+^ is crucial for the environment. There are various methods to detect Fe^3+^, such as fluorescent analysis [3], atomic absorption spectroscopy [4] and inductively coupled plasma analysis [5]. Noteworthily, the fluorescent analysis method has the advantages of simplicity, high sensitivity, and fast response, which meet the need for rapid and accurate detection.

Carbon quantum dots (CQDs) are a new type of fluorescent carbon material with a diameter of less than 10 nm [6,7]. Compared with metal-based quantum dots, CQDs are biocompatible, environment-friendly and low-cost, which makes them a promising alternative in analytical chemistry. Due to their high specific surface area, small particle size and facile surface functionalization [8], CQDs have high activity towards various targets, resulting in changes in their fluorescence. For example, Zhang et al. [9] prepared CQDs by hydrothermal method using black sesame as a carbon precursor. This dual-function fluorescence sensor could detect Fe^3+^ and L-ascorbic acid. However, the quantum yield (QY) of CQDs derived from natural biomass is low and has to be improved. The composition and surface functional groups of CQDs are important factors affecting the luminescence and sensing performance of CQDs, and heteroatomic doping can improve the optical performance and quantum yield of CQDs [10,11]. Xylan is a non-aromatic molecule whose main chain is linked by a beta-(1 → 4) glycosidic bond. China is a big agricultural country, where corn and sugarcane are important crops. As renewable resources, corncob and bagasse contain abundant xylan and are important raw materials for the preparation of xylan. The excellent properties of xylan such as degradability, biocompatibility and renewability have attracted the attention of experts and scholars at home and abroad. Xylan derivatives prepared by certain means can be used in food additives, paper additives, drug carriers, wound dressings, capacitor energy storage devices, degradable films, etc., which broadens the high-value utilization and industrial application of xylan [12,13]. Therefore, xylan is a promising carbon precursor with low cost, abundant resources and renewable energy. We prepared CQDs by heteroatom-doped xylan.

Generally, sensors with a single readout of fluorescent CQDs are inevitably disturbed by ambient temperature, light, state/parameters of the spectrophotometer, operational error, etc. [14]. In order to overcome this reliability barrier, it is a good strategy to adopt a ratio strategy in the design of a fluorescence probe, which simultaneously emits two or more fluorescence signals at different wavelengths and their intensity ratio is measured for quantification. The ratio fluorescence probe provides one inert fluorescence signal as an internal reference for self-calibration [15]. Thus, external effects are eliminated and the reliability as well as sensitivity of the target detection are improved [16]. The red fluorescence of rhodamine B is centered around 572 nm, which does not coincide with the emission wavelength of most CQDs. Meanwhile, due to its relatively stable chemical properties and high stability to various metal ions, rhodamine B with red fluorescence was an appropriate self-reference signal.

However, the traditional fluorescent analysis relies on the lab and cannot conduct on-site, rapid detection. Fortunately, the fast development of smartphone technology in the past decade provides excellent access. For their powerful computing ability, high-resolution camera and customizable software program, convenient on-site inspection is made possible. Meanwhile, paper, a cheap, biodegradable, renewable and portable carrier for fluorescent probes, shows advantages with smartphones among plastic, glass, textile, etc. It has been reported that a variety of smartphone-linked sensors for collecting different types of signals, including optical, impedance and electrochemical current, largely promotes the development of smartphone-based detection platforms [17]. For instance, Liu et al. reported a smartphone-based sensing system for quantitative assays of 2, 4, 6-trinitrotoluene by using printed electrodes to receive impedance signals [18]. Lin and Yu reported a smartphone device to shoot and record the color changes of sensor fluorescence caused by streptomycin, and the corresponding digital images were then transferred to red–green–blue (RGB) data and analyzed by a smartphone [19].

**Scheme 1:**
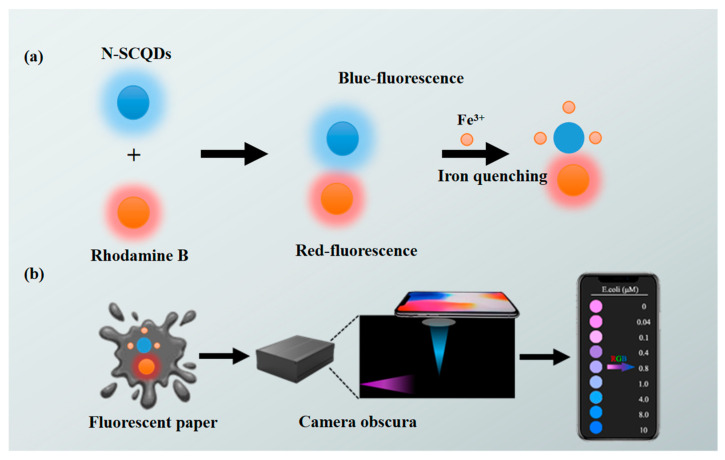
Illustration of (**a**) fabrication of a paper-based platform with rhodamine B–CQD ratio fluorescent probe, (**b**) on-site, visual and rapid detection of Fe^3+^ using smartphone.

Therefore, in this study, a paper-based optical sensor based on a ratio fluorescence probe was established and further combined with a smartphone for visual, on-site and quantitative detection of Fe^3+^ (Scheme 1). CQDs derived from xylan and amikacin sulfate with N, S doping to improve QY functioned as signal response part in the ratio fluorescence probe, where red luminous rhodamine B acted as an internal reference. Further, a fluorescent paper was fabricated by infiltrating circular paper in the ratio probe. The measurement of fluorescent signals depended on a smartphone instead of a fluorescence spectrophotometer. This research establishes a kind of ratio fluorescence sensing platform, showing a broad prospect in the real-time monitoring of metal ions for the environment.

## 2. Results and Discussion

### 2.1. Characterization of N-SCQDs Prepared via Hydrothermal Method

**Figure 1:**
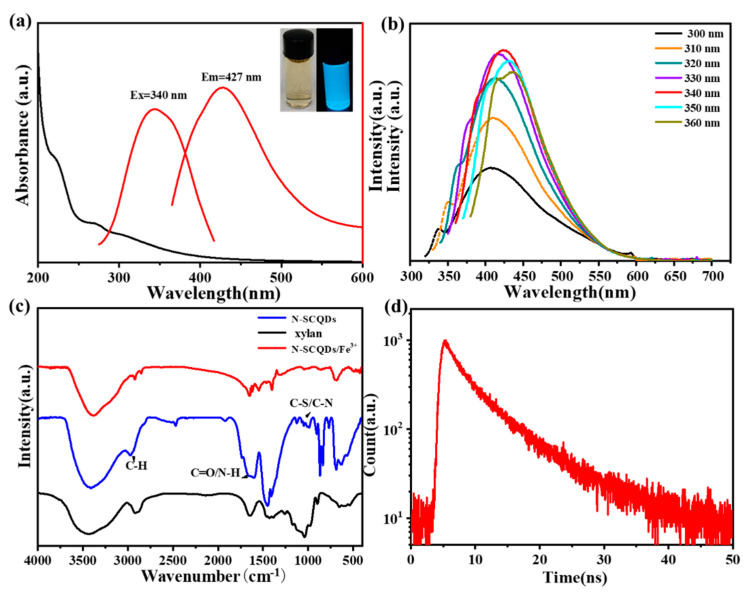
(**a**) Fluorescence excitation, emission and UV−vis spectra of N−SCQDs, the inset is images of N−SCQDs solution under sunlight or UV light (365 nm); (**b**) fluorescence emission spectra of N−SCQDs with different excitation wavelengths; (**c**) FT−IR spectrum of N−SCQD_S_, xylan and N−SCQD_S_/Fe^3+^; (**d**) fluorescence attenuation spectra of N−SCQDs.

As is shown in the inset of Figure 1a, nitrogen-sulfur-doped CQDs (N-SCQDs) emitted bright blue fluorescence under ultraviolet light, and their aqueous solution was light yellow under sunlight. According to UV–vis spectra, N-SCQDs had a characteristic peak at 240 nm, which was attributed to the π-π* transition of aromatic C=C bonds [20].

The fluorescence properties of N-SCQDs were comprehensively characterized by fluorescence excitation, emission and attenuation spectra. As shown in Figure 1a, its fluorescence excitation wavelength was centered around 340 nm, and the excited emission wavelength was 427 nm. The emitted fluorescence was affected by the wavelength of excitation light. With the increase of excitation wavelength, the emitted fluorescence gradually redshifted, and the maximum fluorescence was excited by light of 340 nm (Figure 1b). Using quinine sulfate as the standard, the fluorescence quantum yield of N-SCQD_S_ was 24.6%. Compared with xylan, the functional groups of N-SCQDs were different due to the doping of nitrogen and sulfur elements. According to their FT-IR spectrum (Figure 1c), except for the significant enhancement of the peak at 1408 cm^−1^ (C-OH) and 1687 cm^−1^ (C=O), there was additional peaks near 1205 cm^−1^, which may be attributed to the stretching vibration of C-N and C-S [21]. All these indicated that N-SCQDs contained abundant functional groups. When Fe-trivalent ions were added to N-SCQDs, the peak at 1408 cm^−1^ (C-OH), 875 cm^−1^ (C=C) and 1687 cm^−1^ (C=O) decreased significantly. This may be because the hydroxyl group and the carboxyl group have some chelating effect on the trivalent iron ion. According to the time-resolved fluorescence attenuation curve (Figure 1d), the attenuation curve was fitted as I(t) = 526^(−t/2.27)^ + 396^(−t/7.78)^ [22], it was calculated that the average fluorescence life of N-SCQDs was 6.98 ns.

**Figure 2:**
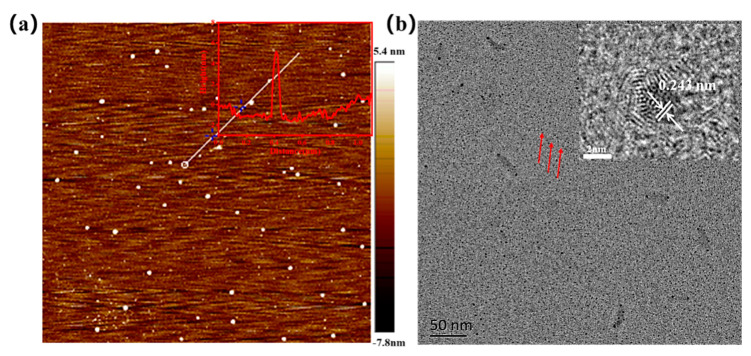
(**a**) AFM image of N-SCQDs and height distribution of the white line region in (**a**). Brown represents the background and white dots represent the quantum dots; (**b**) TEM image of N-SCQDs and HRTEM image of N-SCQDs.

The morphology of N-SCQDs was investigated using AFM and TEM, as shown in Figure 2a,b. N-SCQDs were monodisperse quasi-spherical shaped with a height of 3–5 nm (illustration of Figure 2a), indicating that the prepared N-SCQDs consisted of a single layer or several layers of graphene sheets. In HRTEM images of N-SCQDs (illustration of Figure 2b), the lattice stripes of 0.24 nm corresponding to the (1120) plane of graphite were clearly visible [23], confirming a certain crystallinity of N-SCQDs.

**Figure 3:**
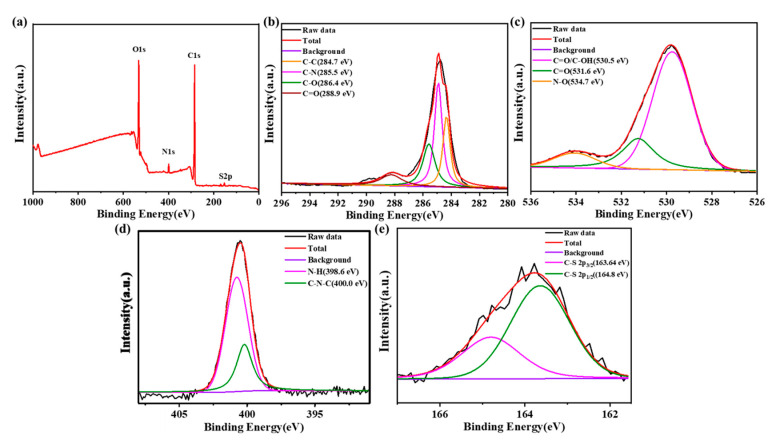
(**a**) XPS spectrum of N-SCQDs; high-resolution XPS spectra of (**b**) C1s, (**c**) O1s, (**d**) N1s and (**e**) S2p.

In the full-scan XPS spectrum (Figure 3a), there were four typical peaks at 164.5 eV, 285 eV, 400 eV and 530.8 eV, assigned to S2p, C1s, N1s and O1s, respectively. The result indicated that after reaction with amikacin sulfate, the N-SCQDs contained nitrogen, sulfur functional groups and abundant oxygen-containing functional groups [24]. Figure 3b is the high-resolution XPS spectrum of C1s. It can be seen that carbon element has four different chemical states C=C, C-N, C-O and C=O, which were at 284.7 eV, 285.5 eV, 286.4 eV and 288.9 eV, respectively [25]. The oxygen-containing functional groups of N-SCQDs were analyzed in the high-resolution XPS spectra of O1s (Figure 3c). The peaks at 530.5 eV, 531.6 eV and 534.7 eV were attributed to C=O/C-OH, C=O and N-O, respectively [26]. In the high-resolution XPS spectra of N1s (Figure 3d), the peaks of 398.6 eV and 400.0 eV are associated with N-H and C-N-C, respectively [24]. Figure 3e is the high-resolution XPS spectrum of S2p, which mainly shows the peak of C-S [21]. These results indicated that the core of doped N-SCQDs is a carbon cluster, and the edge comprises a large number of oxygen-containing functional groups and a small number of nitrogen and sulfur functional groups.

### 2.2. Construction of Ratio Fluorescence Probe for Fe^3+^ Detection

**Figure 4:**
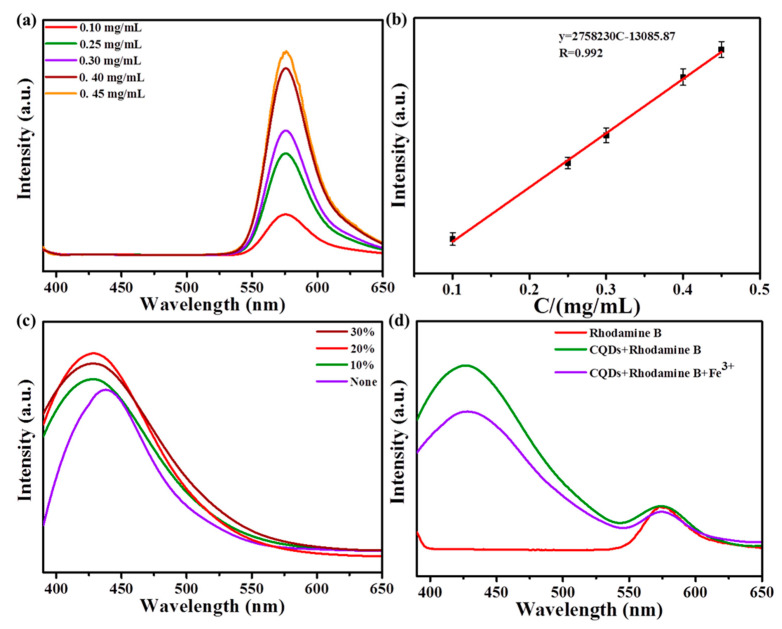
(**a**) Fluorescence spectra of different concentrations of rhodamine B under the excitation of 340 nm; (**b**) linear relationship between the concentration of rhodamine B and fluorescent intensity; (**c**) fluorescent spectra of N−SCQDs prepared with different dosages of amikacin sulfate; (**d**) effect of Fe^3+^ on the fluorescence of rhodamine B and N−SCQDs.

In order to overcome the influence of the environment on fluorescence intensity, the fluorescent dye, rhodamine B, was added to construct the ratio fluorescent probe. Figure 4a shows the fluorescence spectra of rhodamine B with different concentrations at the excitation wavelength of 340 nm. It could be seen that the fluorescence intensity increased with the increase in concentration and had a good linear relationship. As shown in Figure 4b, the linear regression equation was Y = 2.76 × 10^6^C − 1.31 × 10^4^. (R = 0.992) (Y: fluorescence intensity; C: concentration).

During the preparation of N-SCQDs via the hydrothermal method, the effect of the dosage of amikacin sulfate on their fluorescent performance was discussed. As shown in Figure 4c, when amikacin sulfate was not added, the emission peak of carbon quantum dots was at 437 nm. When amikacin sulfate was added, the summit was blue-shifted to 427 nm, indicating that the doped elements N and S had a significant impact on the emission spectrum. In addition, when the dosage of amikacin sulfate was 10%, the fluorescent intensity was enhanced, and when the dosage increased to 20%, the fluorescent intensity was greatly enhanced, but when the dosage was 30%, the fluorescent intensity was weakened. Therefore, N-SCQDs were prepared via hydrothermal method with 20% amikacin sulfate.

As shown in Figure 4d, when only rhodamine B was in solution, there was an obvious emission peak at 572 nm. When rhodamine B and N-SCQDs were mixed, a dual emission system was formed at 427 nm and 572 nm with the same excitation wavelength. After the addition of Fe^3+^, it was found that the emission peak of N-SCQDs decreased significantly, while the peak intensity of rhodamine B basically remained unchanged, indicating that Fe^3+^ can quench the fluorescence of N-SCQDs rather than that of rhodamine B, which provides a possibility for the construction of ratio fluorescent probe for the detection of Fe^3+^.

**Figure 5:**
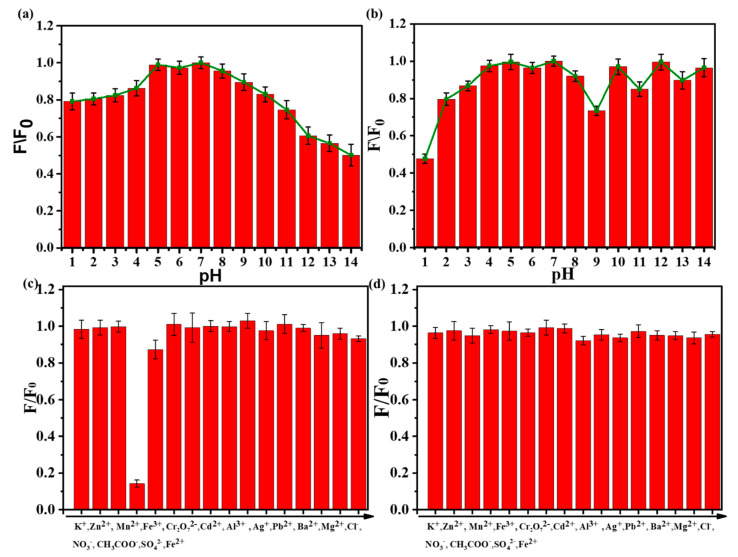
(**a**) Influence of pH on fluorescence intensity of N−SCQDs solution; (**b**) effect of pH on fluorescence intensity of rhodamine B solution; (**c**) detection selectivity of N-SCQDs among different ions (the concentration for interference ion detection is 200 μM); (**d**) effect of various metal ions on the fluorescence of rhodamine B (the concentration for interference ion detection is 200 μM).

The pH value of the solution has a great influence on the fluorescent intensity of N-SCQDs and fluorescent dyes. Therefore, we studied the fluorescence intensity of N-SCQDs and rhodamine B at different pH values. As shown in Figure 5a, in a neutral solution, the fluorescence intensity of N-SCQDs was the strongest. When the pH became acid or alkaline, the fluorescence became weak. This may be caused by the different charges of the surface groups in the acid–base environment. Amino groups are positively charged in acidic environments and carboxyl groups are negatively charged in alkaline environments. The surface effect of functional groups affects the excited radiation transition efficiency of N-SCQDs, and finally leads to different fluorescence intensities [27]. As can be seen from Figure 5b, rhodamine B was almost unaffected by pH. Therefore, considering the influence of pH on N-SCQDs and rhodamine B, the pH value of the solution should be stabilized to neutral to avoid its interference with the detection of Fe^3+^.

### 2.3. Detection of Fe^3+^ Using Ratio Fluorescence Probe

As shown in Figure 5c, the effect of various ions (K^+^, Zn^2+^, Mn^2+^, Fe^3+^, Cr_2_O_7_^2−^, Cd^2+^, Al^3+^, Ag^+^, Pb^2+^, Ba^2+^, Mg^2+^, Cl^−^, NO_3_^−^, CH_3_COO^−^, SO_4_^2−^, Fe^2+^) on the fluorescence of N-SCQDs was discussed. The quenching rate of N-SCQD fluorescence by Fe^3+^ could reach 85.8%, while the quenching rate of N-SCQD fluorescence by other ions was less than 13.2%, indicating that Fe^3+^ had a specific quenching effect on N-SCQD fluorescence compared with other ions, thus N-SCQDs could be used to specifically identify Fe^3+^. Subsequently, the effects of these ions on the fluorescence of rhodamine B were investigated (Figure 5d), and it was found that these ions had little effect on the fluorescence of rhodamine B—their quenching rates were all less than 5%, which provided a good condition for the construction of dual emission ratio fluorescent probes for the detection of Fe^3+^.

**Figure 6:**
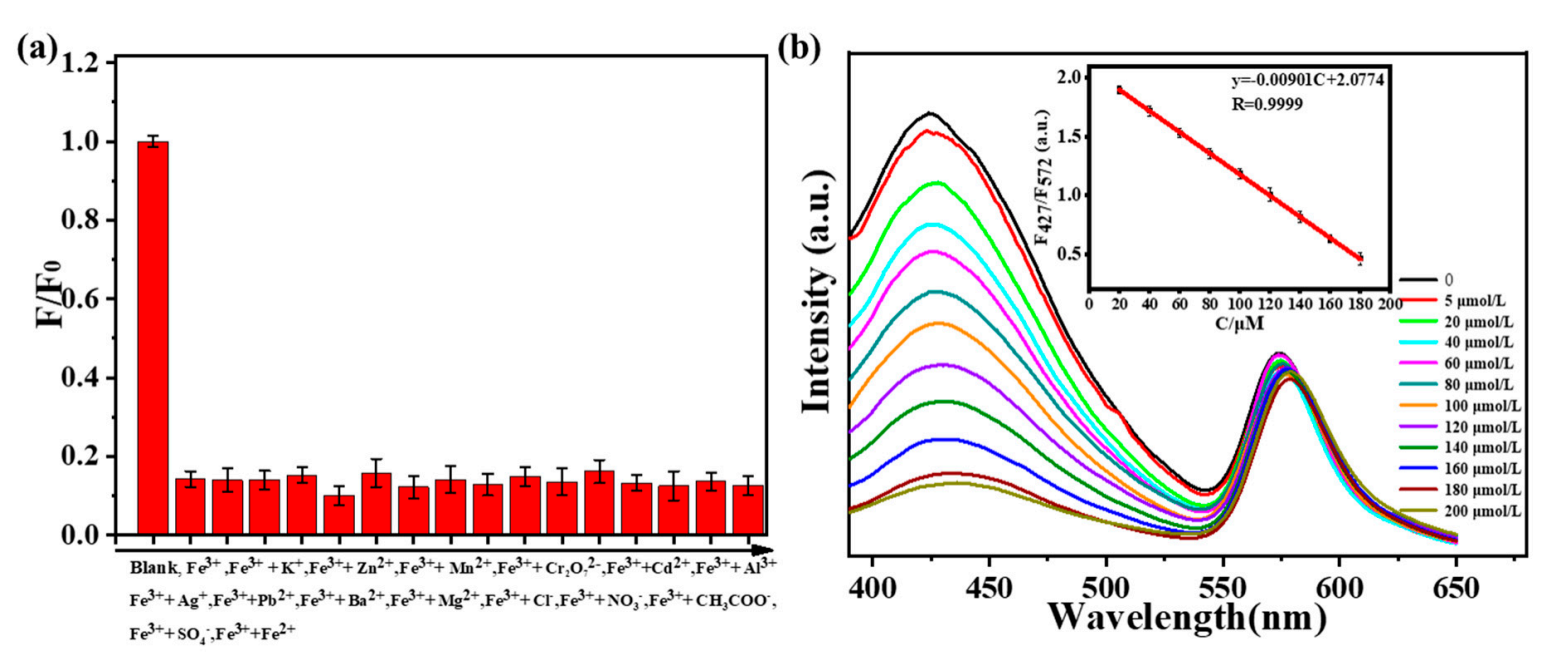
(**a**) Quenching efficiency of Fe^3+^ toward N−SCQDs−rhodamine B under the disturbance of other ions. (**b**) Fluorescence spectra of N-SCQDs/rhodamine B solution excited at 340 nm with different concentrations of Fe^3+^; the linear relationship between the fluorescence intensity of N−SCQDs/rhodamine B solution and Fe^3+^ concentration.

In order to avoid the influence of the environment on fluorescence intensity, a ratio fluorescence probe was constructed to detect Fe^3+^ in solution. As shown in Figure 6a, when one of the ions (K^+^, Zn^2+^, Mn^2+^, Cr_2_O_7_^2−^, Cd^2+^, Al^3+^, Ag^+^, Pb^2+^, Ba^2+^, Mg^2+^, Cl^−^, NO_3_^−^, CH_3_COO^−^, SO_4_^2−^, Fe^2+^) exists, the quenching efficiencies of Fe^3+^ on N-SCQDs–rhodamine B are 86.2%, 86.0%, 84.8%, 90.2%, 84.2%, 87.8%, 85.9%, 87.2%, 85.1%, 86.5%, 83.5%, 86.7%, 87.5%, 86.5% and 87.8%, respectively. The influence of these ions on the quenching efficiency is below 6.7%, indicating that ionic interference on the quenching effect of Fe^3+^ is weak. Different concentrations of Fe^3+^ (0–200 μM) were added to the solution containing N-SCQDs–rhodamine B, and the fluorescence intensity was measured with a fluorescence spectrometer. As shown in Figure 6b, with the increase of Fe^3+^ concentration, the fluorescent intensity at 427 nm (emission peak of N-SCQDs) gradually decreased, while the fluorescence intensity at 572 nm (emission peak of rhodamine B) changed negligibly. In the range of 20–180 μM, there was an excellent linear relationship between Fe^3+^ concentration and the ratio of fluorescent intensity at 427 nm and 572 nm. The linear equation was y = −9.01 × 10^−3^C + 2.08 (R = 0.9999), (y: fluorescence intensity; C: concentration). Furthermore, the detection limit (S/N = 3) was estimated to be 42.3 nM.

### 2.4. Paper-Based Platform for Detection of Fe^3+^ in Water

**Figure 7:**
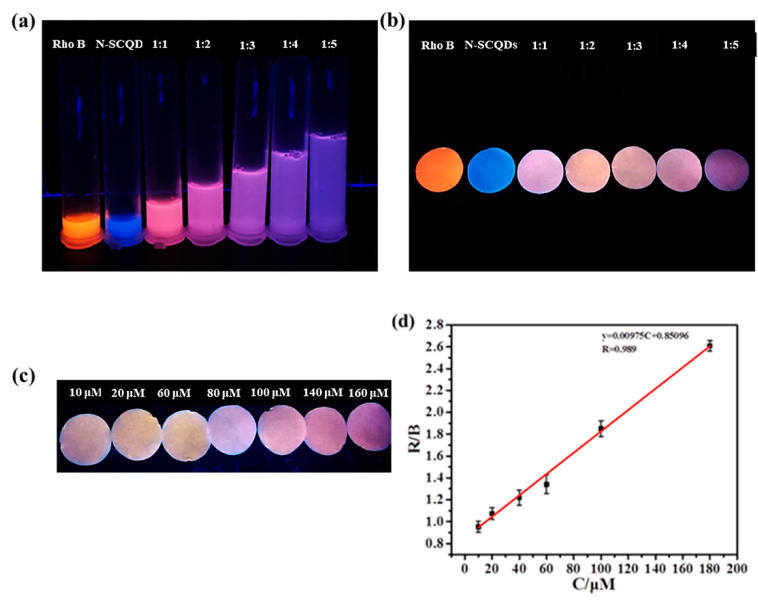
(**a**) Fluorescent images of N-SCQDs–rhodamine B in different proportions under 365 nm UV light; (**b**) fluorescent images of paper dripped with N-SCQDs–rhodamine B solutions under 365 nm UV light; (**c**) the effect of different concentrations of Fe^3+^ on fluorescent paper (rhodamine B: N-SCQDs = 1:2); (**d**) the linear relationship between the chromaticity value of fluorescent paper (R/B) and Fe^3+^ concentration.

Since the ratio fluorescent solution was not convenient enough for detection, the platform combined fluorescent paper with the smartphone was built here to carry out visual and quantitative detection of Fe^3+^. As shown in Figure 7a, pure rhodamine B solution emits bright red fluorescence, while N-SCQDS solution emits bright blue fluorescence. When rhodamine B solution and N-SCQDS solution were mixed in different proportions, it was found that the color of fluorescence changed from pink to dark purple. After soaking in the mixture for 20 min and drying, the fluorescence of the paper under UV light was measured (Figure 7b). It was found that the paper presented similar fluorescence with the solution in Figure 7a. In order to quantitatively detect Fe^3+^ with fluorescent paper, fluorescent paper with rhodamine B and N-SCQDs ratio of 1:2 was chosen for its large response range in color of fluorescence. The fluorescent papers were soaked in Fe^3+^ solution in the concentration range of 0–180 μM for 5 min, and their images taken using a smartphone are shown in Figure 7c. By measuring chroma value, the results showed that with the increase of Fe^3+^ concentration, the R/B (red/blue) value of the fluorescent disk gradually increased. In the range of 10–180 μM, the Fe^3+^ concentration and R/B value were fitted to the equation Y = 9.75 × 10^−3^C + 0.851, and the limit of detection of Fe^3+^ was calculated to be 198.2 nM by dividing the slope of the linear equation by three times the standard variance [28].

### 2.5. Possible Mechanism of Iron Ion Detection

**Figure 8:**
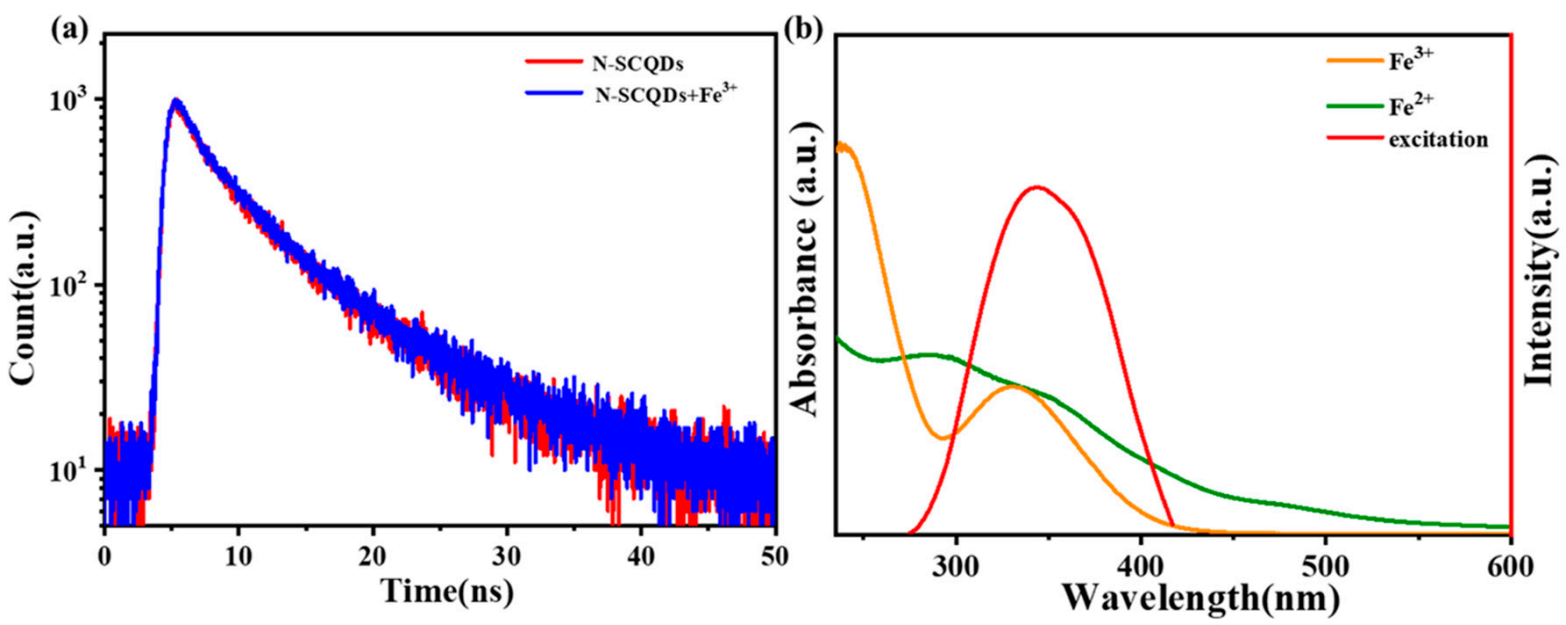
(**a**) Fluorescence attenuation curve of N-SCQDs with/without Fe^3+^; (**b**) fluorescence excitation spectrum of N-SCQDs and UV–vis absorption spectra of Fe^3+^ and Fe^2+^.

We further investigated the quenching mechanism of Fe^3+^ on a ratio fluorescence probe. As shown in Figure 8a, when Fe^3+^ was added to the solution of N-SCQDs, the fluorescence attenuation curve almost overlapped with the curve without Fe^3+^. Its attenuation curve was I(t) = 593exp(−T/2.405) + 387exp(−T/8.0006), and the average fluorescence lifetime was 7.076 ns, which was nearly the same with N-SCQDs. The results indicated that there was no fluorescence resonance effect and dynamic quenching between N-SCQDs and Fe^3+^. According to Beer’s law: A = −logT = −log(I/I0) = εbC (where A is absorbance and ε is molar absorption coefficient or extinction coefficient), the UV absorbance of 200 μM of ferric ion at 340 nm is 0.9. Therefore, it is calculated that ε = 0.0325 in this experiment. Furthermore, the fluorescence excitation spectrum of N-SCQDs and the UV–vis absorption spectra of Fe^3+^ and Fe^2+^ are shown in Figure 8b. Fe^3+^ had a strong absorption peak in 275–415 nm, while the wavelength of the light required for excitation of N-SCQD fluorescence was 265–425 nm; these two ranges overlapped perfectly. Therefore, when Fe^3+^ was added to the N-SCQDs solution, the light required for excitation of N-SCQD fluorescence was absorbed by Fe^3+^, thus making fluorescence quenched, namely, the inner filter effect. When the Fe^3+^ was reduced to Fe^2+^ (absorption wavelength of 225–300 nm), the quenched fluorescence was restored.

**Figure 9:**
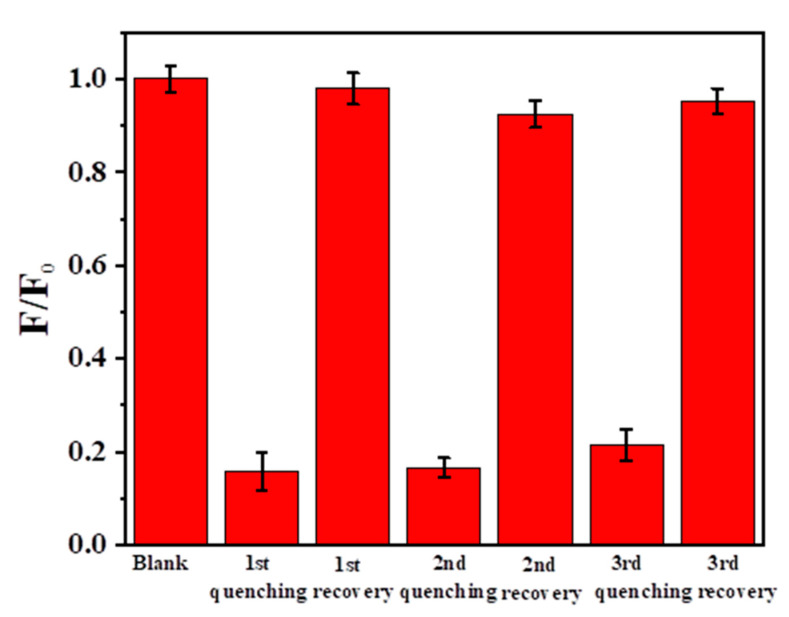
Fluorescence during multiple addition/reduction with Fe^3+^ and ascorbic acid.

Due to the strong destructive ability of the oxidizing agent, the fluorescence of N-SCQDs would also be quenched while oxidizing Fe^2+^ to Fe^3+^. Therefore, the multiple oxidation/reduction of Fe was replaced by addition/reduction, which added additional Fe3+ after reduction instead of oxidation of Fe^2+^ to Fe^3+^. This measure would avoid the influence of oxidation on fluorescence. As shown in Figure 9, when ferric ion was added, the fluorescence of the blank was greatly reduced to 85.8% After the addition of ascorbic acid (100 μM), the fluorescence was almost completely restored. By repeating quenching and recovery three times, the fluorescence still recovered 94.3%, although the degree of fluorescence quenching and recovery gradually decreased. This may be due to that the ascorbic acid added each time is slightly excessive and the chelation with Fe^3+^/Fe^2+^ disrupts fluorescent recovery. This suggests that the sensor could potentially be reused in a limited time.

## 3. Experimental Section

### 3.1. Reagents and Chemicals

Xylan and cellulose dialysis bag (molecular cut off 500 Da) were extracted from bagasse and purchased from Shanghai Yuanye Biotechnology Co., Ltd. (Shanghai, China). Amikacin sulfate and sodium hydroxide were purchased from Shanghai Maclin Biochemical Technology Co., Ltd. (Shanghai, China), and other reagents were common commercially available products. Elf11/6b/301 Muffle furnace was purchased from Verder Shanghai Instrument Equipment Co., Ltd. (Shanghai, China).

### 3.2. Preparation of Nitrogen-Sulfur-Doped CQDs

In total, 0.8 g of xylan and amikacin sulfate were dissolved in 20 mL of sodium hydroxide/urea (8%/12%, *w*/*w*) aqueous solution. The mixture was transferred to a 25 mL Teflon-lined stainless steel autoclave and heated in a muffle oven at 240 °C for 18 h. The obtained mixture was centrifuged at 14,000 rpm for 10 min to remove the precipitate. Nitrogen-sulfur-doped CQDs (N-SCQDs) (0.3 mg/mL) were obtained after one week of dialysis with a 500 Da dialysis bag and stored at room temperature away from light.

### 3.3. Preparation of N-SCQDs–Rhodamine B

N-SCQDs (0.3 mg/mL) and rhodamine B (0.1 mg/mL) were mixed in a ratio of 2:1 and stirred under magnetic stirring for 20 min to obtain N-SCQDs–rhodamine B, which was stored at room temperature away from light.

### 3.4. Material Characterization

The UV–vis spectrum was investigated using a UV-1800 spectrophotometer (Daojin, Shanghai, China). FT-IR spectra were investigated on a Tensor 27 infrared spectrometer (Bruker, Karlsruhe, Germany). TEM images were taken with a JEM-2010F transmittance electron microscope (JEOL, Tokyo, Japan). AFM images were obtained using a NanoScope atomic force microscope (Veeco Metrology Group, Camden, NJ, USA). XPS spectra were measured with an ESCALAB 250 (Themo-VG Scientific, Minneapolis, MN, USA). The obtained XPS peaks were deconvoluted using XPSpeak41. SEM images were obtained using a Merlin field emission 9 scanning electron microscope (Zeiss, Carl Zeiss, Germany).

The FL spectra of N-SCQDs were measured on a fluoroMax-4 fluorescence spectrophotometer (Horiba, Minneapolis, MN, USA).

### 3.5. Effect of pH on Fluorescence of N-SCQDs

Aqueous solutions with pH = 1, 2, 3, 4, 5, 6, 7, 8, 9, 10, 11, 12, 13, 14 were adjusted by 0.1 M hydrochloric acid and 0.1 M sodium hydroxide. 30 μL of N-SCQDs (0.3 mg/mL) was added to 3.0 mL of aqueous solution with a certain pH value. After mixing evenly, fluorescence spectra under excitation of 340 nm were measured by fluoromax-4 fluorescence spectrophotometer.

### 3.6. Effect of Ions on Fluorescence of N-SCQDs and Rhodamine B

MnSO_4_, KBr, Al(NO_3_)_3_, Pb(NO_3_)_2_, K_2_Cr_2_O_7_, CdCl, AgNO_3_, FeCl_3_, BaCl_2_, NaCl, NaNO_3_, CH_3_COONa and Na_2_SO_4_ were chosen as the ion sources of Mn^2+^, K^+^, Al^3+^, Pb^2+^, Cr_2_O_7_^2−^, Cd^2+^, Ag^+^, Fe^3+^, Ba^2+^, Cl^−^, NO_3_^−^, CH_3_COO^−^ and SO_4_^2−^, respectively (the concentration for interference ion detection is 200 μM). A total of 30 μL of N-SCQDS solution (0.3 mg/mL) and 30 μL of ion solution (2 × 10^−2^ M) were added to 3 mL of deionized water, and the fluorescent spectra were measured under the excitation of 340 nm laser. Similarly, 3 mL of deionized water was added with 10 μL of rhodamine B solution (0.1 mg/mL) and 30 μL of ion solution, and the fluorescence spectra were measured under the excitation of 340 nm laser.

### 3.7. Determination of Fe^3+^ Using N-SCQDs–Rhodamine B

In total, 30 μL of N-SCQDs solution (0.3 mg/mL), rhodamine B solution (0.1 mg/mL) and Fe^3+^ solution of different concentrations were added to 3 mL of deionized water. After mixing, fluorescence spectra were measured using a fluoromax-4 fluorescence spectrophotometer.

MnSO_4_, KBr, Al(NO_3_)_3_, Pb(NO_3_)_2_, K_2_Cr_2_O_7_, CdCl_2_, AgNO_3_, FeCl_3_, BaCl_2_, NaCl, NaNO_3_, CH_3_COONa and Na_2_SO_4_ were chosen as the ion sources of Mn^2+^, K^+^, Al^3+^, Pb^2+^, Cr_2_O_7_^2−^, Cd^2+^, Ag^+^, Fe^3+^, Ba^2+^, Cl^−^, NO_3_^−^, CH_3_COO^−^ and SO_4_^2−^, respectively. When these interfering ions are present, 200 μM of ferric ions are added for fluoromax-4 fluorescence spectrophotometer determination and comparison.

N-SCQDs solution (0.3 mg/mL) and rhodamine B solution (0.1 mg/mL) were mixed evenly in a ratio of 2:1. Furtherly, the circular paper (filter paper) with a radius of 1 cm was put into the 3 mL mixture and soaked for 20 min before drying to form fluorescent paper. Then, the fluorescent paper was immersed in 1 mL Fe^3+^ solution of different concentrations for 2 min and then photographed using a mobile phone under a UV lamp. Photoshop 5 was used to determine the RGB value of the picture, so as to quantitatively detect Fe^3+^.

## 4. Conclusions

By mixing amikacin sulfate and xylan as precursors, N- and S-doped carbon quantum dots were obtained after hydrothermal treatment. The addition of amikacin sulfate could effectively change the properties of carbon quantum dots. The ratio fluorescence probe consisting of N-SCQDs and rhodamine B improved the reliability and sensitivity of Fe^3+^ detection. Platform combined fluorescent paper with a smartphone was further fabricated to realize on-site, visual and quantitative detection of Fe^3+^. The linear range of detection was 10–180 μM with a limit of detection of 198.2 nM, and the detection principle was based on the inner filter effect, which effectively quenched the fluorescence. Therefore, the application of ratio fluorescent paper with a smartphone provides a facile method for on-site detection of ions and high-value utilization of agricultural and forestry by-products.

## Data Availability

Data are contained within the article.
